# Enhancing objective structured clinical examination performance through an artificial intelligence virtual patient: a proof-of-concept study

**DOI:** 10.1080/08998280.2026.2699604

**Published:** 2026-07-21

**Authors:** Betty S. Chan, Jack Marjot, Timothy Dodds, Lucy S. R. Jo, Hyun Yang, Jen Xiang, Paul Dyer, Bhavani Kannan, Graham Hannah, Adrienne Torda

**Affiliations:** a School of Clinical Medicine, University of New South Wales, Kensington, Australia; b Emergency Department, Prince of Wales Hospital, Sydney, Australia; c Immersive Technologies, University of New South Wales, Kensington, Australia; d Medicine and Health Education Development Unit, University of New South Wales, Kensington, Australia; e Learning and Teaching Group, UNSW Canberra, University of New South Wales, Kensington, Australia

**Keywords:** Artificial intelligence, feedback, objective structured clinical examination, remediation, virtual patient

## Abstract

**Background:**

The application of artificial intelligence (AI) in simulating detailed patient-doctor interactions for objective structured clinical examinations (OSCEs) remains emerging. This study aimed to evaluate an AI virtual patient (AIVP) innovation designed to support medical education through interactive patient simulations and feedback.

**Method:**

This prospective mixed-methods pilot recruited final-year medical students during their critical care term. Two cohorts were examined: a volunteer AIVP group (n = 43) and an educational-support medical education enhancement training (MEET) group (n = 8). Participants completed pre- and postintervention OSCEs scored across communication, history and examination, management, and overall outcome domains. Students accessed nine emergency medicine scenarios featuring AI-driven dialogue, speech recognition, avatar interaction, and immediate feedback. Quantitative outcomes were analyzed using the Wilcoxon signed-rank test. Postintervention surveys (Likert scale) and focus groups provided qualitative data, which were analyzed thematically.

**Results:**

In the AIVP cohort, descriptive improvements were observed across domains; however, Wilcoxon testing identified no statistically significant changes. MEET students demonstrated significant improvement in overall OSCE outcomes (median 37 [IQR: 30–67] vs 67 [IQR: 47–75]; *P* = 0.03), with positive trends in all domains. Survey responses (n = 25) showed high agreement that AIVP enhanced confidence in history-taking (83%), differential diagnosis (78%), and management planning (78%). Thematic analysis from 10 students demonstrated that AIVP was experienced as a structured, exam-focused, and confidence-building learning tool that addresses gaps in workplace-based learning.

**Conclusion:**

AIVP was feasible and well received, providing practice aligned with university OSCE standards. While the volunteer cohort showed modest gains, the MEET group demonstrated significant improvement, suggesting benefit for students needing structured support. Tailored feedback and alignment with OSCE standards highlight AIVP as a promising adjunct for training.

Artificial intelligence (AI) can now recreate high-fidelity human interactions, including virtual patients in medical settings.^1^ Its use in medical education is emerging but remains in early stages of adoption,^2^ and evidence demonstrating measurable educational outcomes remains limited.^3^

Taking a medical history is a fundamental skill, with an estimated 80% of diagnoses made from history alone.^4^ History-taking involves structured questioning to reach a provisional diagnosis, followed by diagnostic reasoning and explanation of next steps. However, real-patient access for practice is limited; patients may refuse, be too unwell, or there may be insufficient supervision.^5^

Simulated patient-doctor interactions in objective structured clinical examinations (OSCEs) help bridge this gap but are resource intensive and constrained by simulated-patient fatigue and performance variability.^3^ An AI virtual patient (AIVP) offers the potential for consistent yet adaptable clinical encounters with flexible access, supporting independent OSCE practice.^6^

This pilot project evaluated the medical education enhancement training using the AI virtual patient (MEET AIVP) platform in phase 3 (fifth or sixth year) medical students at the University of New South Wales. Two cohorts were examined: a volunteer AIVP group and an educational-support MEET group, comprising students identified as requiring additional support to achieve the expected standard. The study explored whether participation improved communication, history-taking, diagnostic reasoning, and management skills and whether AIVP could function as a scalable, standardized supplement to OSCE preparation using performance data and student perceptions.

## METHODS

This prospective mixed-methods pilot study assessed changes in OSCE performance before and after AIVP use, alongside student perceptions collected via surveys and focus groups. Participants were final-year medical students undertaking their critical care term.

Two recruitment pathways were used. The AIVP cohort comprised volunteers recruited via faculty communications. The MEET cohort included students identified by a senior emergency physician as requiring additional structured preparation based on prior assessments or supervisor feedback during the critical care term. Participation was framed as supportive and developmental.

### OSCE assessment

All participants undertook individual pre- and postintervention OSCE stations assessed by a senior emergency physician. Each station mirrored the final-year summative OSCE. Stations included a 2-minute reading period and 12-minute interaction, assessed across communication, focused history/examination, and investigations, diagnosis, and management. Domains were scored using standard categorical ratings, with an overall score calculated.

### AIVP intervention and platform features

Following the baseline OSCE, participants received independent access to the AIVP platform. Feedback data were available from 29 students: 16 in a first group (August–September 2024) and nine in a second group (January–August 2025). All completed pre-AIVP OSCE, used AIVP during the access period, and then undertook the post-AIVP OSCE and survey. The survey was conducted using a Qualtrics questionnaire comprising Likert-scale and qualitative open-ended questions designed to evaluate students’ perceptions of AIVP as a learning tool for history-taking, clinical reasoning, communication, and feedback *(Supplemental Material)*.

The platform contained nine emergency medicine scenarios authored by senior emergency physicians, including chest pain, shortness of breath, headache, back pain, abdominal pain, fever, vomiting, and overdose. Cases replicated authentic emergency department encounters and required both technical and interpersonal skills.

Built in Unity, the platform used OpenAI Whisper speech-to-text for spoken input, ChatGPT-4 for adaptive dialogue, and text-to-speech synthesis for natural responses. Three-dimensional avatars with distinct personalities and communication styles increased engagement. An AI tutor module provided immediate feedback mapped to OSCE criteria. Students could repeat cases, trial different questioning strategies, and refine their approach in a low-stakes environment.

### Thematic focus group discussion

Students were invited to participate in a focus group discussion using a semi-structured guide that explored students’ prior experiences of history-taking education, perceived strengths and limitations of AIVP, and suggestions for future development and curricular integration *(Supplemental Material)*. Three focus groups were conducted between June and August 2025. All discussions were audio-recorded and transcribed verbatim. Transcripts were deidentified prior to analysis.

### Data analysis

Quantitative OSCE data were analyzed using Wilcoxon signed-rank tests. Survey data were analyzed descriptively. Focus group discussion data were analyzed using reflexive thematic analysis as described by Braun and Clarke.^7^ Analysis proceeded through six iterative phases: familiarization, initial coding, theme development, theme refinement, theme definition, and reporting. Analysis was interpretive and reflexive, with themes understood as analytic constructions rather than objective representations of participants’ views. The research team engaged in ongoing discussion to refine interpretations and ensure transparency of analytic decisions.

## RESULTS

### Quantitative outcomes

From 2024 to 2025, 50 students were recruited; seven dropped out, leaving 43 AIVP participants with complete data. Eight additional students formed the MEET cohort, giving 51 students with paired OSCE results.

In the AIVP cohort, small improvements were observed across all domains, but no statistically significant group-level change was detected. Median overall OSCE scores increased from 63 to 70 (*P* > 0.29) *(Figure 1a)*. Some individuals moved from borderline or fail to pass categories, but numbers were insufficient for statistical significance.

**Figure 1.**
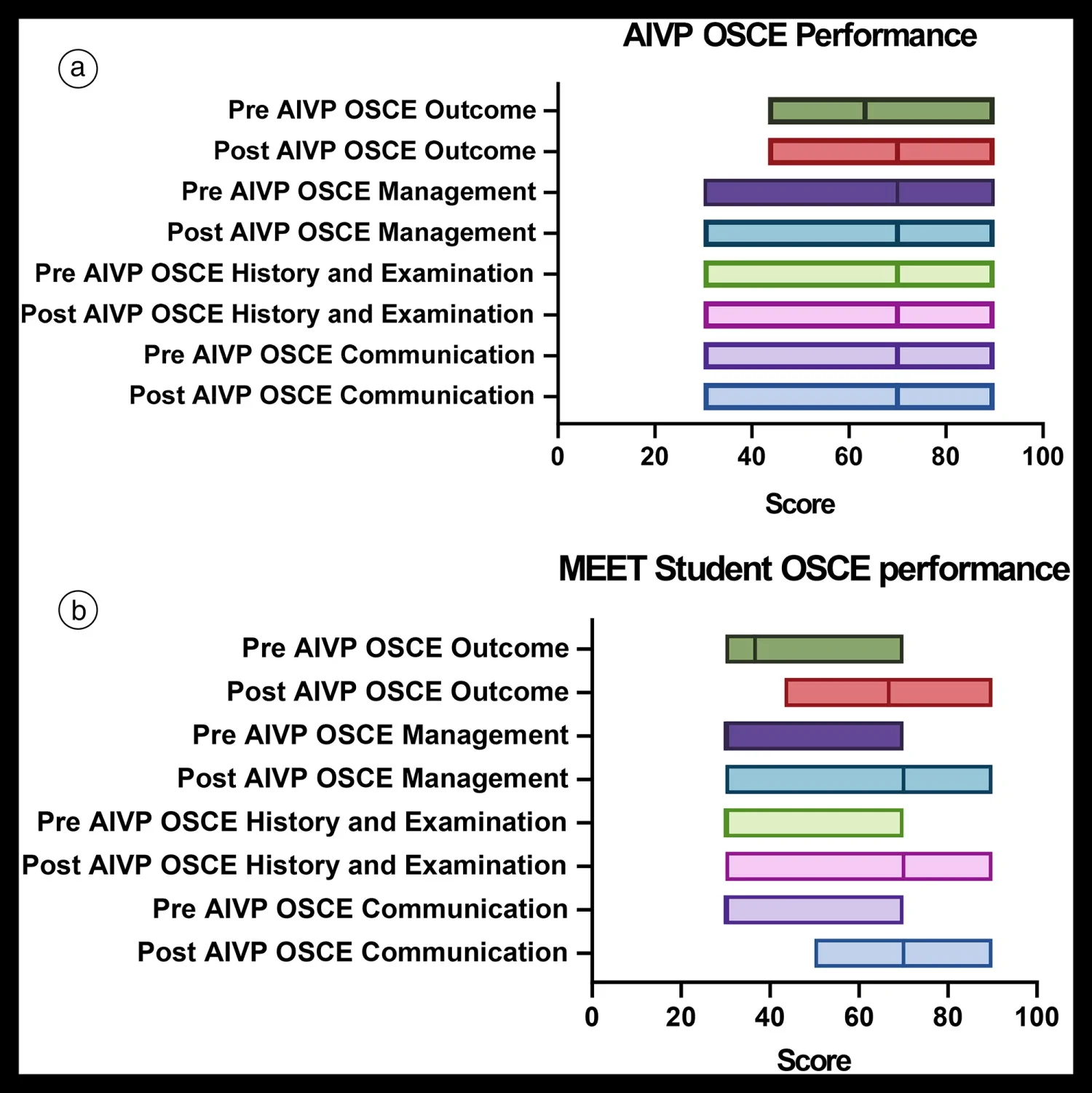
Performance on objective structured clinical examinations (OSCEs) in two cohorts: the **(a)** artificial intelligence virtual patient (AIVP) cohort and **(b)** the medical education enhancement training (MEET) group. The box plots represent the range of results, and the line in the middle represents the median OSCE score for each domain.

The MEET cohort demonstrated larger median improvements across domains. While individual domains did not reach statistical significance, overall OSCE performance improved from a median of 37 to 67 (*P* = 0.03), indicating a significant global gain despite the small sample size *(Figure 1b)*.

### Survey feedback

Most students agreed that AIVP improved confidence in history-taking (83%), asking appropriate questions (89%), discussing differential diagnoses (78%), and outlining management plans (78%). The perceived impact on communication skills was lower (61%). Feedback was rated as useful (75%) and appropriately delivered (69%). While most students felt avatar responses were generally natural (78%), fewer agreed that character traits were fully authentic (67%). Overall, students rated AIVP as a valuable (89%), widely applicable (89%) learning experience that offered opportunities not otherwise available in the curriculum (78%).

### Thematic analysis

Ten students participated in focus groups. Analysis revealed a coherent narrative describing how AIVP addressed gaps in clinical learning, particularly reduced access to structured practice and feedback during placements.

Students described a transition from highly structured early-years teaching to more opportunistic workplace learning. Within this context, AIVP was valued for reintroducing structure, predictability, and exam alignment. Timed cases supported prioritization and decision-making under pressure, reducing OSCE-related anxiety. Integration of history, investigations, and management reinforced clinical reasoning as a continuous process. *Figure 2* summarizes the thematic structure and analytic findings.

**Figure 2.**
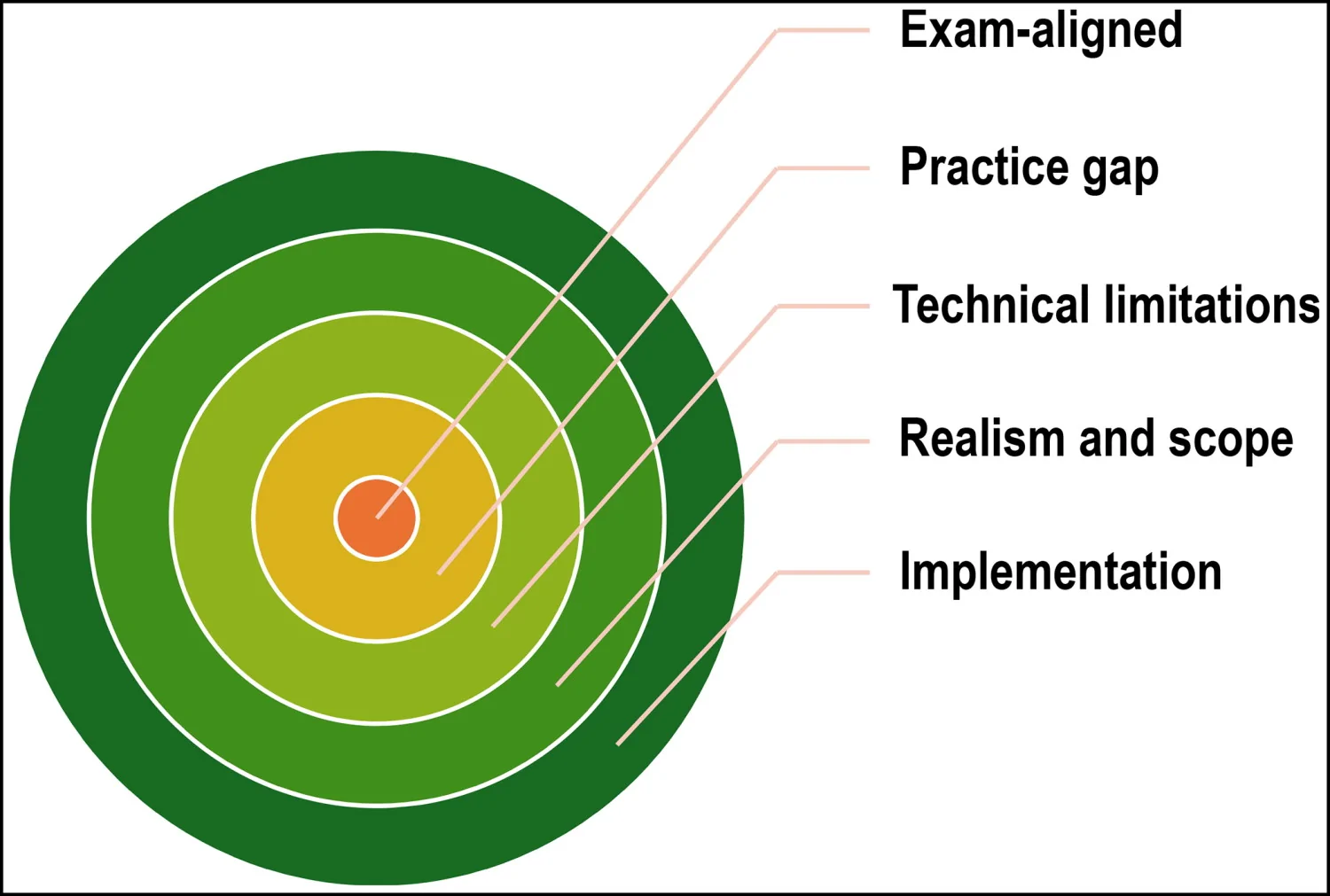
The five themes related to students’ experiences with the artificial intelligence virtual patient (AIVP) identified through reflexive thematic analysis. Exam-aligned structured practice is positioned as the core perceived value of AIVP (orange). Surrounding layers represent factors motivating engagement and shaping expectations (yellow and light green), constraints affecting authenticity and usability (green), and broader curricular considerations related to implementation and workload (dark green).

Feedback was a central strength. Students valued identification of missed high-yield questions and described feedback as more consistent and objective than that typically received on placement, positioning AIVP as both a practice and formative assessment tool.

Technical limitations constrained realism in this iteration. Delayed responses, transcription errors, and occasional role confusion disrupted conversational flow and limited rapport-building. As a result, AIVP was viewed as more effective for cognitive and procedural skills than nuanced interpersonal communication. Despite these limitations, students expressed strong enthusiasm for future development, advocating expansion into other specialties and sensitive scenarios. Careful curricular integration was emphasized, with modest, predictable workloads aligned to existing teaching considered optimal.

## DISCUSSION

This mixed-methods study evaluated the MEET AIVP platform for clinical skills development in final fifth- and sixth-year medical students. By combining OSCE performance data, surveys, and focus group discussions, the study provides a nuanced picture of how AI-driven virtual patients may complement traditional teaching.

While group-level OSCE improvements were modest in the volunteer cohort, significant gains in the MEET cohort and strong student-reported benefits highlight the value of AIVP as a targeted adjunct, particularly for students requiring additional support.^8–10^ Students endorsed AIVP for supporting structured history-taking, diagnostic reasoning, and management planning, consistent with emerging literature on generative AI in medical education.^11–13^

Thematic findings emphasized its role in addressing gaps in workplace-based learning, while also identifying current limitations in interpersonal authenticity, echoing broader simulation research.^14^ From a curricular perspective, findings underscore the importance of integrative implementation. Educational technologies achieve maximal impact when embedded within existing educational structures rather than introduced as add-on interventions.

In conclusion, this study contributes to growing evidence that AI-driven virtual patients can complement traditional clinical education. AIVP appears most effective as an exam-aligned tool supporting cognitive and procedural skill development, particularly in remediation contexts. Future work should prioritize technical refinement, improved conversational realism, and thoughtful curricular integration.

## Supplementary Material

Supplemental Material
