# Supplementary material for: Enhancing objective structured clinical examination performance through an artificial intelligence virtual patient: a proof-of-concept study
Source: Proc (Bayl Univ Med Cent). 2026 Jul 21;39(6):1092–5. doi: 10.1080/08998280.2026.2699604 (PMC13689783; doi:10.1080/08998280.2026.2699604)
Supplement: Supplementary file 1 [file UBMC_A_2699604_SM0879.docx]

**Supplemental Material**

**Enhancing objective structured clinical examination performance through an artificial intelligence virtual patient: a proof-of-concept study**

Betty S. Chan, MBBS, PhD, Jack Marjot, MBBS, Timothy Dodds, BA, Lucy S. R. Jo, PhD, Hyun Yang, Jen Xiang, MEnvResEc, Paul Dyer, BCs, Bhavani Kannan, LLB, MDipl, Graham Hannah, MDM, and Adrienne Torda, MBBS, PhD

***Baylor University Medical Center Proceedings* 2026;39(5)**

## **Appendix A. Artificial Intelligence Virtual Patient (AIVP) Questionnaire**

We invite you to participate in an innovative research project here at UNSW, exploring the potential of an Artificial Intelligence Virtual Patient (AIVP) to enhance medical training. This new study is designed specifically for suitable senior medical students like you, who are currently in their 5th or 6th year and completing their Critical Care Term. This study explores the ability of an AI virtual patient to enhance technical and non-technical skills training for medical students. It also aims to evaluate the AI virtual patient’s effectiveness in providing feedback for both medical students and Objective Structured Clinical Examinations (OSCEs) examiners.

Please note that your involvement is completely voluntary and will not impact your course grades or assessments in any way. Whether or not you choose to participate, your academic standing will remain unaffected.

Submission of your completed questionnaire constitutes your consent for your responses to be used in the research study.

**Please rate your agreement with the following:**

**Participating in the AIVP trial:**

| Increased my confidence in my ability to take a history from a patient. |  |
| --- | --- |
| Improved my ability to ask appropriate questions in a medical history. |  |
| Improved my ability to communicate effectively with a patient. |  |
| Improved my ability to discuss a likely diagnosis / differential diagnosis with a patient. |  |
| Improved my ability to discuss next steps in assessment and management with a patient |  |

**The AIVP:**

| Exhibited authentic character traits |  |
| --- | --- |
| Answered questions in a natural manner |  |
| Stayed in the role of the patient the whole time |  |
| Responded well to student errors or mistakes |  |
| Provided answers that were sufficient to allow me to formulate a differential diagnosis |  |
| Provided me with useful feedback on my performance at the end of the interaction |  |
| Delivered feedback in a style that was conducive to my learning |  |

**Overall, the use of the AIVP:**

| Was a valuable learning experience |  |
| --- | --- |
| Did not require a high level of technological skill |  |
| Provided a learning opportunity not met by other aspects of the course |  |
| Is a tool that should be used more widely in my medical education |  |

**Please provide any free-text comments to the following:**

**What were the strengths of the AIVP as an educational tool?**

**How could the AIVP, in its role as a virtual *patient*, be improved?**

**How could the AIVP, in its role in *giving* *feedback*, be improved?**

**Overall, what elements of the AIVP most contributed to your excitement and/or engagement as a learner?**

**Are there any other comments you wish to share with us?**

## **Appendix B: Focus Group Questions**

**Research Study Title:** MEET-AIVP: MEDICAL EDUCATION ENHANCEMENT TRAINING USING AN ARTIFICIAL INTELLIGENCE VIRTUAL PATIENT

*Participants will be thanked for joining the focus group and reminded that their participation* *is completely voluntary, and they may withdraw at any time without consequence but that once the focus group commences, it will not be possible to remove their individual contributions to the focus group recordings and analysis.* *Confirmation of consent forms will be undertaken. Participants will be informed prior to the commencement of the audio recording.*

1. As a medical student, do you find taking clinical history to be easy or challenging? – how have you experienced teaching in clinical history-taking in medical school thus far? *What was your previous exposure to the subject area?*

2. You have completed a trial using an AI Virtual Patient (AIVP) to practise interacting with a patient to take a medical history – how did you find this? *What did you enjoy? What did you find difficult or confusing?*

3. To what extent do you feel the interaction was realistic? *Was the language appropriate or inappropriate? Were the responses fluid and of appropriate speed? Did the AIVP volunteer too much or too little information in response to your questions? Did the AIVP deviate from its role of the patient (except when giving feedback)?*
4. The AIVP was designed to give you feedback on your interactions with the virtual patient – how did you find this? *What was useful? What did you find difficult or confusing? How did you apply the feedback provided to further your learning?*
5. How did you find the AIVP compared to other modes of learning you have encountered in your studies? *Was it engaging? Did it help you improve both technical and non-technical skills or one in particular?*

6. How did you find the process of using the AIVP from the perspective of the user experience? *Did you find the platform easy to access and use? Were you able to easily navigate the experience?*

7. Are there other areas of your medical education for which you would find it helpful to interact with an AIVP?

8. Do you have any suggestions for improving this learning experience?
